# Effect of antibiotic prophylaxis for preventing infectious complications following impacted mandibular third molar surgery. A randomized controlled trial

**DOI:** 10.4317/medoral.24274

**Published:** 2021-10-27

**Authors:** Nicolás Yanine, Nicole Sabelle, Valentina Vergara-Gárate, Josefina Salazar, Ignacio Araya-Cabello, Alonso Carrasco-Labra, Conchita Martin, Julio Villanueva

**Affiliations:** 1 Department of Oral and Maxillofacial Surgery, Faculty of Dentistry, University of Chile, Santiago, Chile; 2 Maxillofacial Surgery Unit, San Borja Arriarán Clinical Hospital, Chile; 3 Maxillofacial Surgery Unit, Alemana Clinic of Santiago, Chile; 4 Department of Evidence Synthesis and Translation Research, Science and Research Institute, American Dental Association, Chicago, IL, USA; 5 Department of Oral and Craniofacial Health Science, School of Dentistry, University of North Carolina at Chapel Hill, NC, USA; 6 BIOCRAN (Craniofacial Biology) Research Group, University Complutense, Madrid, Spain

## Abstract

**Background:**

The objective of this study was to determine the effect of antibiotic prophylaxis in preventing postoperative infections after extraction of impacted mandibular third molars.

**Material and Methods:**

A Parallel-group, randomized, blind, placebo-controlled trial was performed. 154 patients were randomly allocated to 2 groups; experimental (n=77) receiving 2g amoxicillin 1 hour prior to surgery and control (n=77) receiving placebo. Primary outcome was postoperative infections and secondary outcome was the need for rescue analgesia.

**Results:**

4.5% of patients developed postoperative infections, five patients of the control group (4 alveolar osteitis, 1 surgical site infection) and two of the experimental group (1 alveolar osteitis, 1 surgical site infection). Difference between groups was not statistically significant, RR=0.4 (95%CI 0.08-1.99, 𝘱=0.41) NNTB=26. Rescue analgesia intake was significantly higher in the control group (41 vs 18 patients of experimental group) RR=0.49 (95%CI 0.32-0.75, 𝘱<0.05) NNTB=3.

**Conclusions:**

The use of 2g amoxicillin 1 hour before surgery was not effective in significantly reducing the risk of postoperative infections from impacted mandibular third molars extraction, when compared to placebo. Nevertheless, antibiotic prophylaxis was associated with a reduced need for rescue analgesia.

** Key words:**Antibiotic prophylaxis, third molar, tooth extraction, impacted tooth, dry socket, surgical wound infection, oral surgery.

## Introduction

The surgical extraction of mandibular third molars is the most common procedure in oral and maxillofacial surgery worldwide. These type of tooth are completely or partially unerupted and positioned against another tooth, bone or soft tissue in a way that its further eruption is unlikely (1). This alteration in the eruption is mainly due to the fact that third molars are the last group of teeth to complete their formation, root development and eruption, and they do so on a defined continent, the dentoalveolar skeletal structure of the maxilla. Postoperative complications of this procedure include pain, edema, trismus, fever, dry socket, and purulent drainage. These complications have an impact on patients' lives, potentially affecting the normal development of daily activities, such as eating, speaking, and maintaining oral hygiene (2). Surgical site infection is one of the most frequent postoperative complications after the extraction of an impacted mandibular third molars (IMTM) (3). The frequency of these postoperative complications varies in the literature between 0% and 16% (4-10).

The surgical site infection is triggered when there is a quantitatively and qualitatively significant bacterial threat. The risk of infection varies according to the type of procedure, the presence of active local infection, the surgical time, and the patient's underlying conditions, such as obesity, decompensated diabetes, or immunosuppression. The frequency of these postoperative complications varies in the literature between 0% and 16% (4-10).

The prevention of surgical site infection should focus on reducing the number of bacteria in the surgical wound and improving the patient's immune response.

A widespread practice among dentists is the use of antibiotic prophylaxis (AP), which consists in the administration of antibiotics before surgery to significantly reduce infection and minimizing adverse effects (2). However, its indication is still widely debated (11). A recent evidence summary on the use of antibiotics in oral and maxillofacial surgery found conflicting results from both clinical trials and systematic reviews, regarding their effectiveness as prophylaxis for third molar extraction (12). Furthermore, systematic reviews have failed to reach consensus due to several factors, one of which is the serious methodological flaws of the included trials (13,14).

The objective of this study was to determine the effect of antibiotic prophylaxis, compared to placebo, in reducing the risk of postoperative infections in patients undergoing impacted mandibular third molar extraction. A secondary objective was to compare the need of rescue analgesia between both groups.

## Material and Methods

- Trial design

The study design was a randomized, parallel-group, blinded and placebo-controlled clinical trial, with a 1:1 assignment of participants to arms. This trial is registered with the Australian New Zealand Clinical Trials Registry, number ACTRN12617001498381 and follows the recommendations from the Consolidated Standards of Reporting Trials (CONSORT) statement (15).

- Participants

Patients with ages ranging between 15 to 35 years, with no comorbidities, that presented at the Maxillofacial Surgery Department of San Borja Arriarán Hospital in Santiago de Chile between the years 2017 and 2019, with at least one clinically and radiographically IMTM with its crown partially covered by mucosa, in position B, class II according to the Pell and Gregory classification (16), were included. Pregnant women, immunocompromised subjects, patients with allergy to penicillin and its derivatives or to nonsteroidal anti-inflammatory drugs (medically diagnosed or patient-reported allergy), patients with gastric ulcer, or that were on antibiotics in the last 30 days before surgery, or those who had an episode of pericoronitis up to 7 days before the intervention were excluded. This study was approved by the institutional ethical review board of the corresponding service. Written informed consent was obtained from all patients.

- Interventions

Patients were allocated either to an experimental group to receive 2g amoxicillin (four 500mg capsules, Andrómaco/Grünenthal. Santiago, Chile) one hour before the third molar extraction or to a control group, which received placebo (four capsules, Cruz Verde Pharmacy Laboratory. Santiago, Chile) one hour before surgery. All procedures were conducted by 3 maxillofacial surgeons with at least 2 years of experience in an ambulatory oral surgery operating room. All patients had only one third molar removed per session. The surgical protocol was as follows: local anesthesia was performed using the Spix technique and superficial cervical plexus block with two cartridges of 1.8 ml of 2% lidocaine with epinephrine (1:100,000) each, per surgical site. A full thickness triangular mucoperiosteal flap (semi Neumann) was created, osteotomy and/or tooth sectioning were performed as conservative as possible, using a low speed round bur and abundant irrigation with 0.9% saline. Osteotomy was classified as mild (⅓ of dental crown height), moderate (⅔ of dental crown height or first third of the root) or major (over ⅔ of dental crown height or second third of the root). Straight elevators were used for every extraction. After the complete extraction of the molar, the excision of pericoronal hyperplastic tissue and alveolar socket conditioning were performed. Immediately after, the socket was profusely irrigated with saline 0.9% for 30 seconds. Silk 3.0 suture was used for the surgical wound closing, without the additional use of any intra or extra alveolar dressing or antiseptic. Finally, postoperative written instructions were explained to all patients (Appendix 3). All patients received a prescription for 400mg ibuprofen Tablets and 500mg paracetamol Tablets every 8 hours for 3 days as anti-inflammatory medication and postoperative analgesic. When patients needed additional pain management or they reported experiencing pain greater than 4 when using a 0 to 10 visual analogue scale, 125 mg lysine clonixinate Tablets were prescribed as rescue analgesia, repeating the dose according to the requirements of each patient.

- Outcomes

The primary outcome was the presence of postoperative infectious complications, described as alveolar osteitis or surgical site infection. Alveolar osteitis was defined as postoperative pain inside and around the extraction site that increased in severity between the first and third day after extraction, along with a partially or completely disintegrated blood clot in the alveolar socket, with or without halitosis (17). Assessment was performed by in-person clinical examination. This process was performed by 2 calibrated researchers (kappa 0,95) who participated exclusively in this phase of the study. Postoperative controls were performed at 3-, 7- and 30-days post-surgery.

Surgical site infection was adjudicated when the patient presented at least one of the following: A: Purulent drainage from the surgical wound or abscess. B: Isolation of pathogenic microorganisms in liquid or tissue cultures from the surgical site. C: Spontaneous dehiscence of the incision site in patients who exhibit at least one of the following signs or symptoms: 1) fever (>38°C), 2) pain from palpation or spontaneous, 3) localized swelling, facial erythema or local heat. D: Severe pain after a week, together with moderate or severe intraoral inflammation and/or moderate or severe intraoral erythema with no other apparent cause, that improves with antibiotic treatment (18).

The secondary outcome was the need of rescue analgesia. Rescue analgesia was defined as the need for an additional dose of analgesic was necessary for the management of persistent pain, and this did not replace or delay the next dose of the first prescribed analgesic. The rescue medication used was lysine clonixinate in 125 mg Tablets.

We also evaluated adverse reactions related to the use of amoxicillin, defined as follows: 1) Allergic reaction: urticaria and/or angioedema, 2) Anaphylaxis: throat or tongue swelling and/or respiratory symptoms. 3) Gastrointestinal reactions: nausea, vomiting, abdominal pain and diarrhea. Assessment was performed by anamnesis during clinical examinations.

- Sample size

A priori sample size was calculated assuming a 15% infection rate in the control group and 2% in the experimental group (type I error: 0.05 and 80% power), and a potential 10% of withdrawal or lost to follow-up. Based on this we estimated that we would need to recruit 154 patients in total.

- Randomization and allocation concealment

Randomization was performed in permuted blocks through a computer-generated list of random numbers (Stata V11.0). The placebo capsules were the same size, color and texture as the amoxicillin capsules and allocation concealment was performed using sequentially numbered and identical containers, which were labelled and prepared in an external facility that kept the randomization sequence concealed from the investigators and clinicians while enrolling participants. Each container was labelled with an additional code to identify the chosen side for the surgery (left or right third molar). In patients presenting with more than one IMTM, the tooth to be extracted between either side was also randomly selected.

- Blinding

Both the surgeon and the patients remained blind for the duration of the study. Outcome assessor, data collector, statistician, health care institution and clinical trial monitor were also blind to the participants’ study arm.

- Statistical methods

The difference in proportions between groups was calculated using the chi-square test with a significance level of 5% (SPSS Statistics V22.0). To determine the magnitude of the effect of the intervention in the outcomes of interest, absolute (number needed to treat for benefit NNTB) and relative measures (relative risk) were calculated along with their 95% confidence interval.

In the event of detecting crossing over of participants from one arm of the study to the other, Intention-to-treat analysis was planned. In the case of withdrawals or lost to follow-up during the course of the trial, methods using a plausible range from more (e.g., worst case scenario) to less stringent results were applied to evaluate the robustness of the findings to missing participant data.

A test for interaction (subgroup analysis) was planned performing a multivariate analysis to determine the extent to which smoking (cigarettes or cannabis sativa), alcohol consumption, operative time, osteotomy, the need for tooth sectioning (need of tooth sectioning vs no need for tooth sectioning) may associated with infectious complications. These analyses used baseline exposure assessments, intraoperative findings and were restricted to participants with nonmissing subgroup data at baseline.

## Results

A total of 154 patients entered this clinical trial and were randomly allocated to two groups. No substantial differences were identified among participants baseline characteristics between arms. (Table 1).

**Figure 1:**
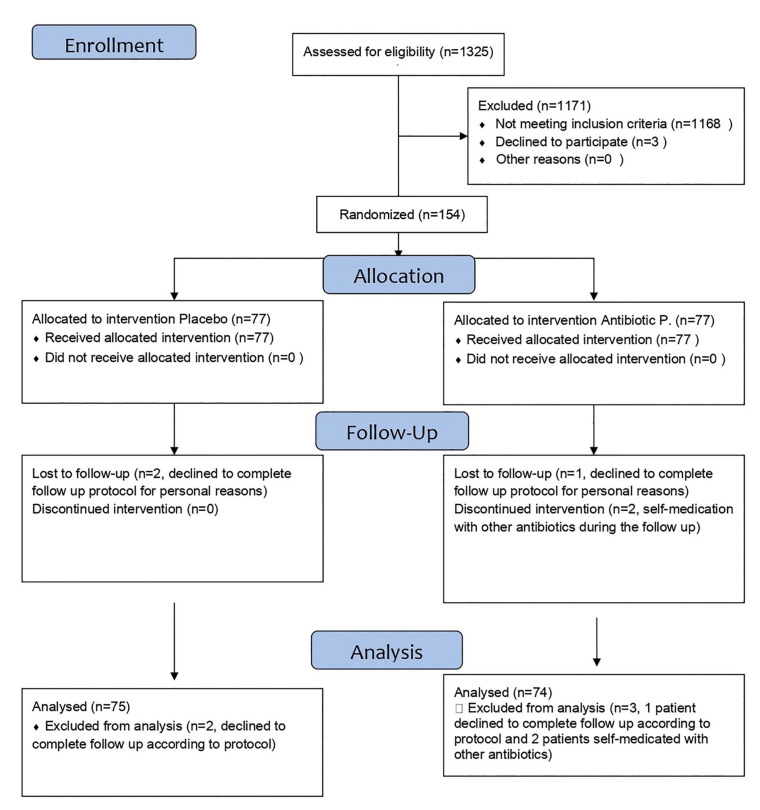
CONSORT Flow Diagram.

**Figure 2:**
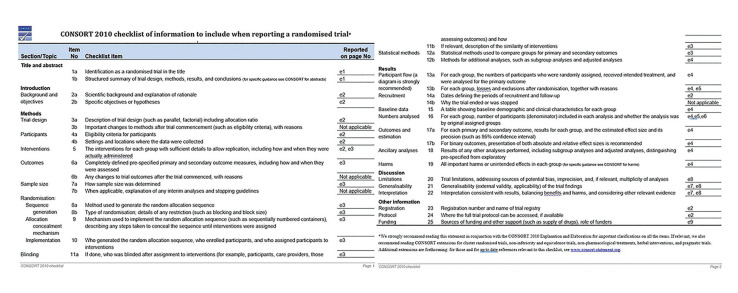
CONSORT checklist of information to include when reporting a randomised trial.

Three patients declined to complete follow up due to personal reasons (2 from the control group and 1 from the experimental group). Two patients were excluded because of self-medication with antibiotics during the follow up period without signs of infection. (CONSORT Flow Diagram, Fig. 1 and Fig. 2).

**Table 1:**
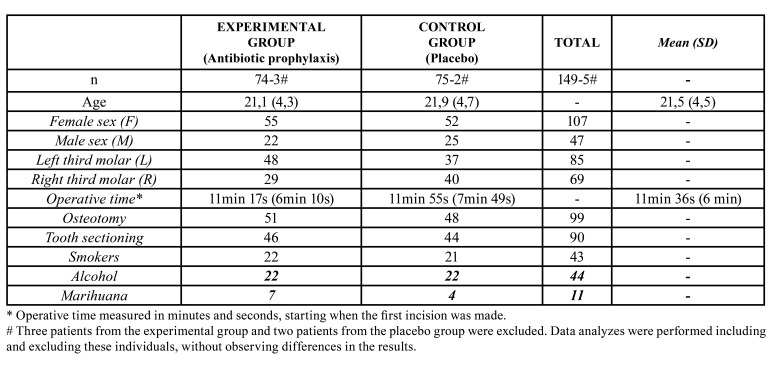
Clinical and demographic characteristics.

None of these patients had postoperative infectious complications at the time of exclusion and when performing best-case and worst-case scenario analysis, results did not vary.

Postoperative infections occurred in 4.5% of all patients studied (n= 154 patients). Table 2 summarizes the characteristics of these patients. Five of them were in the control group: 4 presented alveolar osteitis and 1 presented surgical site infection that manifested as submucosal abscess. In the experimental group, 2 patients presented postoperative infectious complications: one alveolar osteitis and one surgical site infection, also manifested as submucosal abscess (Table 3). The risk reduction of infectious complications was not significantly different between antibiotic prophylaxis group and placebo group. (RR=0.4 95%CI 0.08-1.99, *p*=0.41).

For the secondary outcome, 41 patients of the control group required rescue analgesia (all within the first week after surgery), while in the experimental group only 18 patients needed it (Table 4). Patients receiving antibiotics prophylactically (2 grs of amoxicillin) experienced a 51% reduction in the need of rescue analgesia compared to those in the control arm. RR=0.49 (95%CI 0.32-0.75, *p*=0.03) and NNTB=3.

**Table 2:**
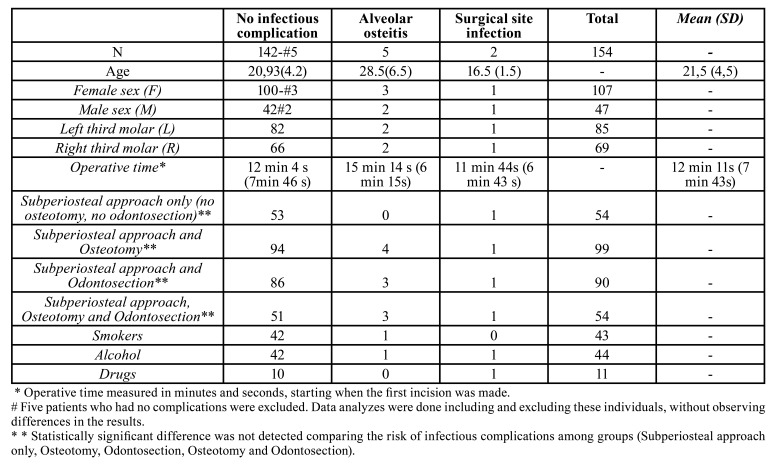
Characteristics of the patients who presented infections.

**Table 3:**
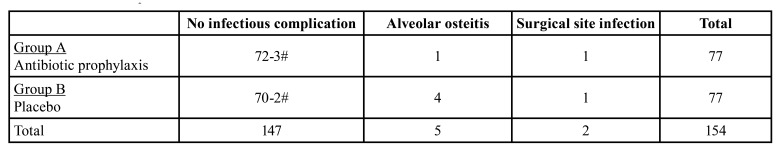
Infectious complications.

**Table 4:**
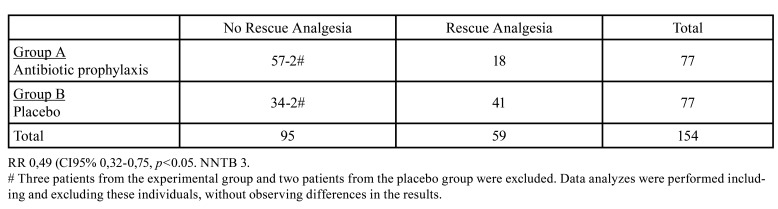
Rescue Analgesia.

Smoking (cigarettes or cannabis sativa) and alcohol consumption were not associated with an increased risk of infectious complications or with the use of rescue medication. Neither were operative times, degree of osteotomy or need of tooth sectioning. None of the patients reported adverse drug reactions during the study period.

## Discussion

In the present trial, participants undergoing IMTM extraction and receiving AP seemed to not experience less postoperative complications compared to those in the control arm, however the requirement for rescue medication associated with postoperative pain in the AP group decreased.

On the incidence of postoperative infections, Lang *et al*. (19) reported 5.7% of infectious complications in a sample of 2,954 patients and Reiland *et al*. (20) reported a 8.5% in a sample of 1,895 patients that underwent third molar extraction at the Mayo Clinic in Rochester. These studies provide a good epidemiological approximation due to the large sample size, which is difficult to achieve in a clinical trial.

In the present study, of the total of patients included, 4.5% presented infectious complications. This percentage is similar to a recent trial that recruited 118 patients and reported a 5,9% of postoperative infections (21). However, among clinical trials there is great variability ranging from 0% to 16% (4-10).

The difference in postoperative complications between groups in this trial was not statistically significant, with 6.5% of the control group and 2.6% of the experimental group presenting infectious complications. A 2015 clinical trial showed similar results, with an incidence of 8.6% in the control group and 3.3% in the experimental group, also without significant difference between groups, but unlike our study, this only included intraosseous third molars, which could decrease the risk of infection when compared to third molars that are communicated with the oral cavity (21). A 2017 study reported an incidence of infectious complications of 5% in the group receiving antibiotics and 7.5% in the control group (20). These Figures are similar to the present study, however, there are several reports where the group that did not receive antibiotic prophylaxis reached an incidence of infections greater than 12% (2,9,22).

All the systematic reviews published up to date suggest that antibiotic prophylaxis may be effective for reducing postoperative complications when extracting mandibular third molars (3,13,23,24). However, most of the primary studies included in these reviews presented serious limitations of study design, which made them prone to risk of bias. More recent trials with better methodological quality and greater statistical power, have failed to find significant results to support the routine use of antibiotics to prevent infectious complications in healthy patients. (21,25).One possible reason to explain the heterogeneity of results in the literature could be the suboptimal reporting and variability in outcome definitions with regards to infectious complications. Another possible reason is the presence of confounding factors such as the use of intra and extra alveolar antiseptics as co-interventions (4,7). Lastly, many of the studies do not specify if there are differences between groups regarding the position of the third molar, which is a factor that could have an important role in the incidence of infectious complications (4).

These reasons could explain this controversy, with some authors recommending its use to prevent infectious complications (2,4,5,7) and others reporting that not only is not effective, but it may be harmful, associating its use with an increase in hypersensitivity and adverse reactions, together with more costs for patients (6,9,26,27).

Regarding the use of rescue analgesia, 23% of the patients in the experimental group compared to 53% the control group required it. This translates into a 51% decrease in the risk of need of rescue analgesia in the group that received AP (RR=0.49 95%CI 0.32-0.75, *p*<0.05), or expressed in another way, for every 3 patients treated with AP, the benefit of avoiding the use of rescue analgesia in one additional patient is obtained. Other randomized clinical trials also reported a significant decrease in postoperative pain and a reduced need for rescue analgesia in patients who received AP (2,7,28). A non-randomized clinical trial conducted Grossi *et al*. in 2007 reported that patients who do not receive antibiotics are at twice the risk of complications associated with postoperative pain (29). On the other hand, a series of reports found no association between the use of AP and reduction in postoperative pain, edema or trismus (6,9,26,27).

The association between the use of antibiotics and the improvement of postoperative symptoms could be explained by the decrease in bacterial contamination of the surgical wound. This in turn would decrease inflammatory mediators, reducing postoperative pain without necessarily affecting the proportion of postoperative infections (30).

Lysine clonixinate was used as rescue analgesia since it can be taken as an additional dose for the management of persistent pain without modifying the analgesic scheme initially prescribed. This prostaglandin inhibitor is rapidly absorbed and takes about 60 minutes to achieve optimal plasma concentration. The drugs used in the primary analgesic and anti-inflammatory scheme in this clinical trial were paracetamol and ibuprofen, both widely used and with sufficient evidence to justify their use in the management of postoperative pain and edema after third molar extraction. The measurement of postoperative edema was not within the objectives of this clinical trial.

For the rest of the variables analysed such as osteotomies, tooth sectioning, operative times, side and position of the third molar, we did not find associations between them and the risk of postoperative infection (Table 2).

The results obtained from this trial showed that the use of 2g amoxicillin one hour before surgery was not effective in reducing the risk of postoperative infectious complications of IMTM extraction in healthy patients.

The patients who received antibiotics presented significantly less postoperative pain, however the administration of antibiotic prophylaxis is not justified for this purpose. Future research should be focused on if an adequate preoperative analgesic scheme may achieve similar results in postoperative pain experience.

In conclusion, the use of 2g amoxicillin 1 hour before surgery was not effective in significantly reducing the risk of postoperative infectious complications from IMTM extraction, when compared to placebo. The use of antibiotic prophylaxis was associated with a reduced need for rescue analgesia.

- Strengths and limitations

The present study was conducted following high standards of methodology to control possible sources of bias and the protocol was designed carefully to prevent methodology issues reported in other publications.

We obtained a smaller difference in infection rates between groups than what we assumed initially when sample size was calculated. This resulted in a wide confidence interval and could explain why antibiotic prophylaxis was not able to demonstrate significant differences.
